# Supplementary material for: Tmsb10 triggers fetal Leydig differentiation by suppressing the RAS/ERK pathway
Source: Commun Biol. 2022 Sep 15;5:974. doi: 10.1038/s42003-022-03941-5 (PMC9478096; doi:10.1038/s42003-022-03941-5)
Supplement: Supplementary file 2 — Supplementary Information [file 42003_2022_3941_MOESM2_ESM.pdf]

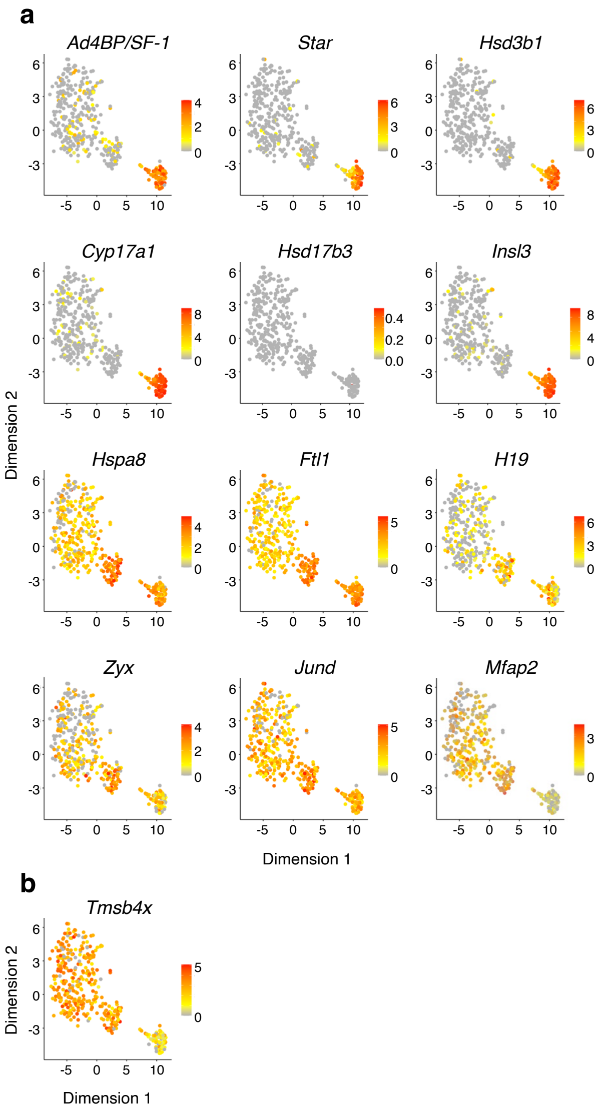

Supplementary Figure 1. Expression profile of the genes classified into group-I to III

Expression profiles of *Ad4BP/SF-1*, *Star*, *Hsd3b1*, *Cyp17a1*, and *Hsd17b3*, and *Insl3* genes in group-I*, Hspa8*, *Ftl1*, and *H19* genes in group-II, and *Zyx*, *Jund*, *Mfap2* genes in group-III are shown in ***(a)***. As described previously, Hsd17b3 required for testosterone production is not expressed in FLCs (Shima et al., 2013). *Tmsb4x* is shown in ***(b)****.* A dot represents a cell.

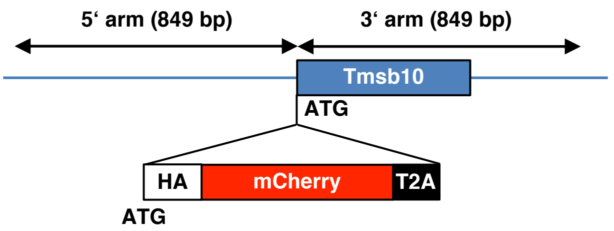

Supplementary Figure 2. Construction of *Tmsb10-mCherry* knock-in plasmid

Construction of the *Tmsb10-mCherry* knock-in plasmid is illustrated. Preparation of the 5’ and 3’ arms and mCherry reporter gene tagged with human influenza hemagglutinin (HA) and Thoseaasigna virus 2A (T2A) are described in ‘Materials and Methods’.

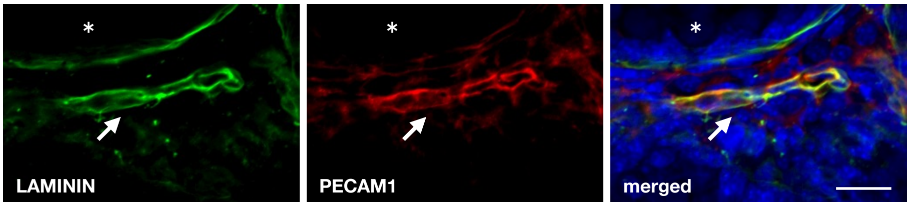

Supplementary Figure 3. Colocalization of LAMININ and PECAM1 in endothelial cells of mouse fetal testis

FLE-EGFP mouse fetal testes at E16.5 were subjected to immunostaining. Immunofluorescence images of LAMININ (green) and PECAM1 (red), and merged image (merged) are shown. Arrows indicate LAMININ and PECAM1 double-positive endothelial cells. Asterisks mark testis tubules. Scale bar = 20 μm.

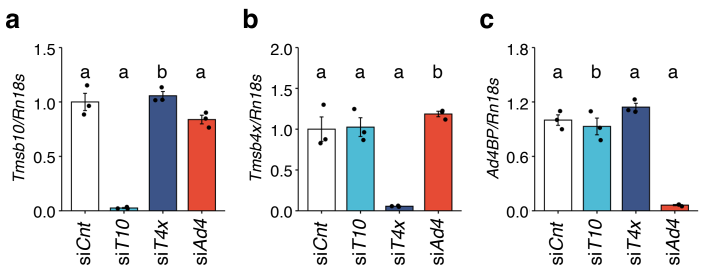

Supplementary Figure 4. Knockdown efficiencies by siRNA treatments

W-EGFP cells were prepared from *FLE-EGFP* fetal mouse testes at E16.5 and cultured as described in ‘Materials and Methods’. They were treated with siRNA for control (si*Cnt*, open bars), *Tmsb10* (si*T10*, light blue bars), *Tmsb4x* (si*T4x*, dark blue bars), and *Ad4BP/SF-1* (si*Ad4*, red bars). The expressions of *Tmsb10* ***(a)***, *Tmsb4x* ***(b)***, and *Ad4BP/SF-1* ***(c)*** in the cells were examined by qRT-PCR. The data were normalized by *Rn18s* and presented as means ± SEM. The different letters denote significant differences between the cell groups. n = 3. p<0.001.

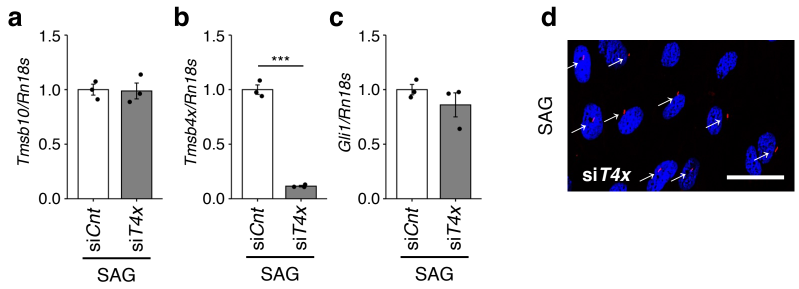

Supplementary Figure 5. Effects of *Tmsb4x* KD on *Gli1* gene expression and ciliogenesis in W-EGFP cells

(a-c) W-EGFP cells were prepared from *FLE-EGFP* mouse fetal testes at E16.5 and cultured as described in 'Materials and Methods'. They were treated with siRNA for *Tmsb4x* (si*T4x*, gray bars) or control siRNA (open bars). Expressions of *Tmsb10* ***(a)***, *Tmsb4x* ***(b)***, and *Gli1* ***(c)*** in the cells were examined by qRT-PCR. The data are standardized using *Rn18s*. n = 3. ***p<0.001. ***(d)*** Expression of ARL13B (red), a cilial marker protein, in the W-EGFP cells treated with si*T4x* was examined by immunostaining. Arrows indicate primary cilia. Nuclei were stained with DAPI (blue). Arrow indicates primary cilia. Scale bar = 10 μm.

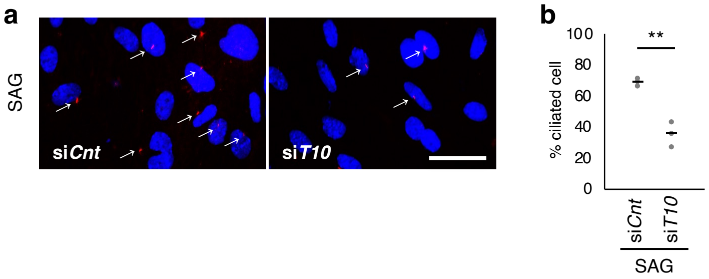

Supplementary Figure 6. Affected ciliogenesis in W-EGFP cells by *Tmsb10* KD in the presence of SAG

(a) W-EGFP cells were treated with si*T10* or si*Cnt* in the presence of SAG. The presence of primary cilia was examined by immunostaining for ARL13B (red). Nuclei were stained with DAPI (blue). Arrows indicate primary cilia. Scale bar = 10 μm. (b) Ciliated cells detected in the studies above were counted. Ratios of the ciliated cells are shown. n=3. **p<0.01.

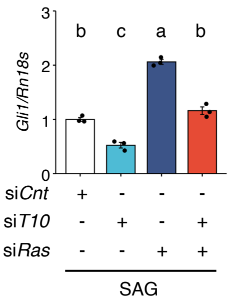

Supplementary Figure 7. *Gli1* expression in W-EGFP cells by *Tmsb10* KD and *Ras* KD in the presence of SAG

W-EGFP cells were treated with si*Cnt* (open bar), si*T10* (light blue bar), si*Ras* (dark blue bar), or both si*T10* and si*Ras* (red bar), and then cultured in the presence of SAG. The expression of *Gli1* was examined by qRT-PCR. The data were standardized using Rn18s. n = 3. $p<0.001$. Letters a, b, and c on the bars denote significant differences between the cell groups.

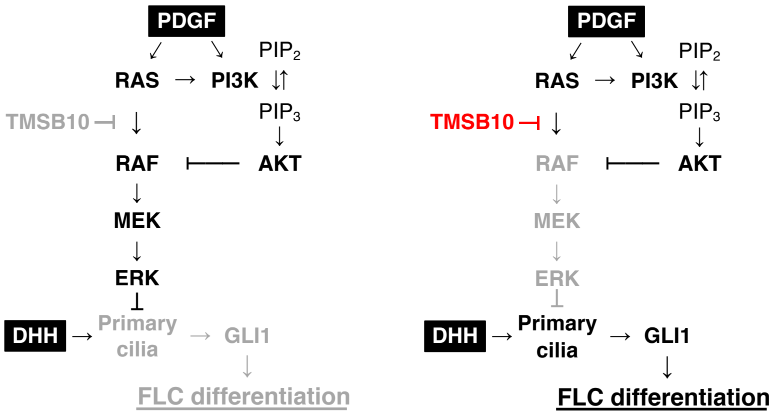

Supplementary Figure 8. Schematics of signal pathways regulating FLC differentiation and in which Tmsb10 acts as a suppressor

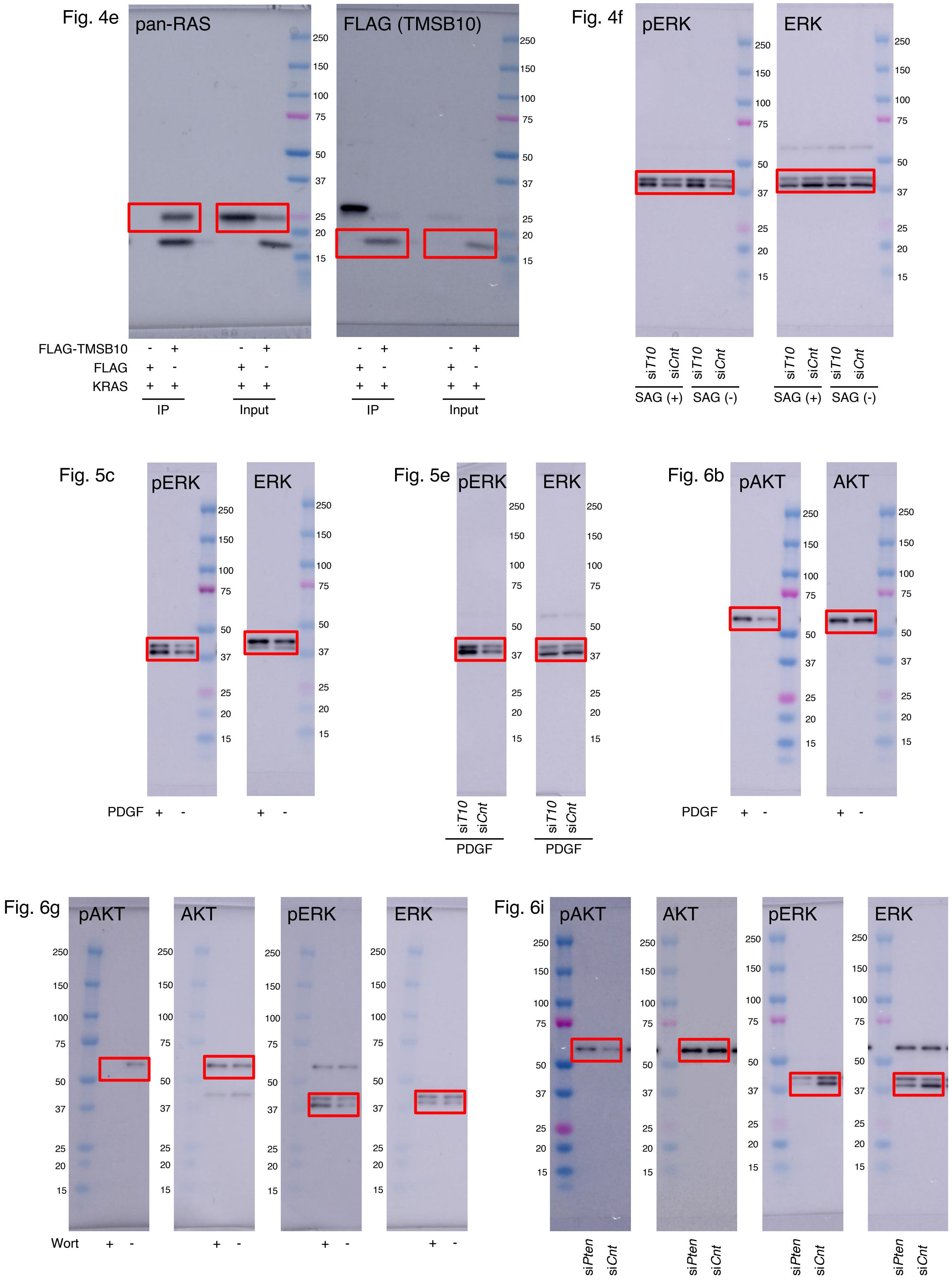

Supplementary Figure 9. Full images of key western blotting

Supplementary Table 1. Quality check of scRNA-seq datasets

	Sequenced cells	Analyzed cells	Total reads	Mean reads/cell
S-EGFP	92	80	22,278,747	242,160
W-EGFP	696	341	103,395,085	148,556

Supplementary Table 2. Primers used for CEL-Seq2 analyses

	Primer (5’ → 3’)
1s	GCCGGTAATACGACTCACTATAGGGAGTTCTACAGTCCGACGATCNNNNNNAGACTCTTTTTTT<br>TTTTTTTTTTTTTTTTV
3s	GCCGGTAATACGACTCACTATAGGGAGTTCTACAGTCCGACGATCNNNNNNAGCTCATTTTTTT<br>TTTTTTTTTTTTTTTTV
8s	GCCGGTAATACGACTCACTATAGGGAGTTCTACAGTCCGACGATCNNNNNNCACTAGTTTTTTT<br>TTTTTTTTTTTTTTTTV
9s	GCCGGTAATACGACTCACTATAGGGAGTTCTACAGTCCGACGATCNNNNNNCAGATCTTTTTTT<br>TTTTTTTTTTTTTTTTV
10s	GCCGGTAATACGACTCACTATAGGGAGTTCTACAGTCCGACGATCNNNNNNTCACAGTTTTTTT<br>TTTTTTTTTTTTTTTTV
18s	GCCGGTAATACGACTCACTATAGGGAGTTCTACAGTCCGACGATCNNNNNNTCGACATTTTTTT<br>TTTTTTTTTTTTTTTTV
25s	GCCGGTAATACGACTCACTATAGGGAGTTCTACAGTCCGACGATCNNNNNNGTTGCATTTTTTT<br>TTTTTTTTTTTTTTTTV
29s	GCCGGTAATACGACTCACTATAGGGAGTTCTACAGTCCGACGATCNNNNNNACCATGTTTTTTT<br>TTTTTTTTTTTTTTTTV
32s	GCCGGTAATACGACTCACTATAGGGAGTTCTACAGTCCGACGATCNNNNNNACGTACTTTTTTT<br>TTTTTTTTTTTTTTTTV
35s	GCCGGTAATACGACTCACTATAGGGAGTTCTACAGTCCGACGATCNNNNNNCTAGACTTTTTTT<br>TTTTTTTTTTTTTTTTV
39s	GCCGGTAATACGACTCACTATAGGGAGTTCTACAGTCCGACGATCNNNNNNCTCAGATTTTTTT<br>TTTTTTTTTTTTTTTTV
44s	GCCGGTAATACGACTCACTATAGGGAGTTCTACAGTCCGACGATCNNNNNNTGCAACTTTTTTT<br>TTTTTTTTTTTTTTTTV
48s	GCCGGTAATACGACTCACTATAGGGAGTTCTACAGTCCGACGATCNNNNNNTGTCGATTTTTTT<br>TTTTTTTTTTTTTTTTV
57s	GCCGGTAATACGACTCACTATAGGGAGTTCTACAGTCCGACGATCNNNNNNAGTGAGTTTTTTT<br>TTTTTTTTTTTTTTTTV
61s	GCCGGTAATACGACTCACTATAGGGAGTTCTACAGTCCGACGATCNNNNNNCAACCATTTTTTT<br>TTTTTTTTTTTTTTTTV
71s	GCCGGTAATACGACTCACTATAGGGAGTTCTACAGTCCGACGATCNNNNNNTCTGTCTTTTTTT<br>TTTTTTTTTTTTTTTTV
72s	GCCGGTAATACGACTCACTATAGGGAGTTCTACAGTCCGACGATCNNNNNNGTCTTCTTTTTTT<br>TTTTTTTTTTTTTTTTV
75s	GCCGGTAATACGACTCACTATAGGGAGTTCTACAGTCCGACGATCNNNNNNGTGAAGTTTTTTT<br>TTTTTTTTTTTTTTTTV
77s	GCCGGTAATACGACTCACTATAGGGAGTTCTACAGTCCGACGATCNNNNNNACAGGATTTTTTT<br>TTTTTTTTTTTTTTTTV
83s	GCCGGTAATACGACTCACTATAGGGAGTTCTACAGTCCGACGATCNNNNNNCTTCTGTTTTTTT<br>TTTTTTTTTTTTTTTTV
88s	GCCGGTAATACGACTCACTATAGGGAGTTCTACAGTCCGACGATCNNNNNNTGGTTGTTTTTTT<br>TTTTTTTTTTTTTTTTV
91s	GCCGGTAATACGACTCACTATAGGGAGTTCTACAGTCCGACGATCNNNNNNGAAGTGTTTTTTT<br>TTTTTTTTTTTTTTTTV
96s	GCCGGTAATACGACTCACTATAGGGAGTTCTACAGTCCGACGATCNNNNNNGAGTGATTTTTTT<br>TTTTTTTTTTTTTTTTV

Supplementary Table 3. siRNA used for KD analysis

Target protein	Reference	
si*Tmsb10*	SASI_Mm01_00030122	Sigma-Aldrich
	SASI_Mm02_00327896	Sigma-Aldrich
si*Tmsb4x*	SASI_Mm01_00163777	Sigma-Aldrich
	SASI_Mm01_00163779	Sigma-Aldrich
si*Ad4BP/SF-1*	SASI_Mm01_00031635	Sigma-Aldrich
	SASI_Mm01_00031636	Sigma-Aldrich
si*Ras* (si*Kras*)	SASI_Mm01_00165654	Sigma-Aldrich
si*Ras* (si*Nras*)	SASI_Mm02_00319691	Sigma-Aldrich
si*Akt* (si*Akt1/2*)	Akt1/2siRNA (m)	Santa Cruz Biotechnology, sc-43610
si*Pten*	SASI_Mm02_00315033	Sigma-Aldrich
	SASI_Mm01_00152659	Sigma-Aldrich

Supplementary Table 4. Primary and secondary antibodies, and their dilution ratios used for immunostaining

Primary antibodies

Target protein	Source	Reference	Dilution
EGFP	Chicken polyclonal	Abcam (Cambridge, UK), ab13970	1:1000
mCherry	Mouse monoclonal	Abcam, ab125096	1:200
LAMININ	Rabbit polyclonal	Sigma-Aldrich, L9393	1:1000
PECAM1	Rat monoclonal	BD Biosciences (San Jose, CA, USA), 557355	1:1000
ARL13b	Mouse monoclonal		1:500

Second antibodies

Target protein	Source	Reference	Dilution
Chicken IgY	Goat anti-chicken IgY H&L (ALEXA Fluor® 488)	Abcam, ab150169	1:500
Mouse IgG	ALEXA Fluor® 555 goat anti-mouse IgG (H+L)	Thermo Fisher Scientific, A21424	1:500
Rabbit IgG	Goat anti-rabbit IgG H&L (ALEXA Fluor® 647)	Abcam, ab150083	1:500
Rabbit IgG	ALEXA Fluor® 488 goat anti-rabbit IgG (H+L)	Thermo Fisher Scientific, A11008	1:500
Rat IgG	ALEXA Fluor® 555 goat anti-rat IgG (H+L)	Thermo Fisher Scientific, A21434	1:500

Supplementary Table 5. Primers used for qRT-PCR analyses

Gene	Forward (5' → 3')	Reverse (5' → 3')
Rn18s	CCATTCGAACGTCTGCCCTAT	GTCACCCGTGGTCACCATG
EGFP	TATATCATGGCCGACAAGCA	TGTTCTGCTGGTAGTGGTCG
Tmsb10	AAGCCGGACATGGGGGAAAT	GTTCAATGGTCTCTTTGGTCGG
Pecam1	ACCAGTCCCCGAAGCAGCACT	GTGGAGCAGCTGGCCTGGAC
Tmsb4x	TCCTCTGCCTTCAAAAGAAACAAT	AGAAGGCAATGCTCGTGGAA
Ad4BP/SF-1	AAGCCACTCTGTAGGACCAAGC	TGTAAATCTGACGCGAAAGCAG
Gli1	ACACAAGTGCACGTTTGAAGG	TCTCATTGGAGTGGGTCCGA
Kras	AGATGTGCCTATGGTCCTGGTAG	CAATCTGTACTGTCGGATCTCTCTC
Nras	CAAGGACAGTTGACACAAAGC	TGTCTTACTACATCAGCACACAG
Akt1	ATGAACGACGTAGCCATTGTG	TTGTAGCCAATAAAGGTGCCAT
Akt2	ACGTGGTGAATACATCAAGACC	GCTACAGAGAAATTGTTCAGGGG
Pten	AATTCCCAGTCAGAGGCGCTATGT	GATTGCAAGTTCCGCCACTGAACA

Supplementary Table 6. Primary and secondary antibodies, and their dilution ratios used for immunoprecipitation and western blotting analysis

Primary antibodies

Target protein	Source	Reference	Dilution
pan-RAS	Mouse monoclonal	Santa Cruz Biotechnology (Lake Placid, NY, USA), sc-32	1:2000
FLAG	Mouse monoclonal	Sigma-Aldrich; F1804	1:5000
pERK	Mouse monoclonal	Santa Cruz Biotechnology, sc-7383	1:2000
ERK	Rabbit polyclonal	Cell Signaling Technology (Danvers, MA, USA), 4695	1:2000
pAKT	Rabbit polyclonal	Cell Signaling Technology, 4060	1:2000
AKT	Rabbit polyclonal	Cell Signaling Technology, 4691	1:2000

Horseradish peroxidase-conjugated second antibodies

Target protein	Source	Reference	Dilution
Mouse IgG	Goat anti-mouse IgG	PerkinElmer Biosciences (Boston, MA, USA), NEF822001EA	1:5000
Rabbit IgG	Donkey anti-rabbit IgG	GE Healthcare, NA9340V	1:5000

Supplementary Table 7. Primers used for construction

Expression plasmid

Gene	Forward (5' → 3')	Reverse (5' → 3')
Tmsb10 (HindIII/BglII)	ACAAAGCTTATGGCAGACAAGCCGGA CATG	ACAAGATCTTTAGGAGATTTCACTCCT CT
Kras (SalI/NotI)	ACAGTCGACCATGACTGAGTATAAAC TTGT	ACAGCGGCCGCTCACATAACTGTACA CCTTG

Donor plasmid

Gene	Forward (5' → 3')	Reverse (5' → 3')
Tmsb10_HA1/2 (SalI/EcoRI)	ATAGTCGACAGAAGGAGACTCTGGAG AGCAAACG	ATAGAATTCCGCATTAAGGGCGAAGC AACTGAG
mCherry (EcoRI/BglII)	ACAGAATTCGGATGGTGAGCAAGGGC GAGGA	ACAAGATCTCTACTTGTACAGCTCGTC CA
Tmsb10_HA1	ATAGTCGACAGAAGGAGACTCTGGAG AGCAAACG	ATCGTATGGGTACATTTTCTTACAACT CTGATGGGCGAAACAAGGTGA
HA-mCherry-T2A	ATGTACCCATACGATGTTCC	ACGTCACCGCATGTCAGCAGACTGCC TCTGCCCTCCTTGTACAGCTCGTCCA TGC
Tmsb10_HA2	GACATGCGGTGACGTGGAAGAGAATC CCGGCCCTATGGCAGACAAGCCGGA CATG	ATAGAATTCCGCATTAAGGGCGAAGC AACTGAG

Tmsb10 guide RNA (gRNA)

gRNA (5' → 3')
CCATCAGAGTTGTAAGAAAATGG

gRNA preparation

Tmsb10 gRNAtemp (5' → 3')
CTAATACGACTCACTATAGCCATCAGAGTTGTAAGAAAAGTTTTAGAGCTAGAAATAGCA

Genotyping

	Forward (5' → 3')	Reverse (5' → 3')
mCherry F1 /Tmsb10 R1	CGCCGACATCCCCGACTACTT	TCTTCAGCTTGGCCTTATCG
Tmsb10 F2/R2	ACCTCCGCCCTTAGACAATG	GTGCGTGAAAGGTTAAGAGG
