# Supplementary material for: Tmsb10 triggers fetal Leydig differentiation by suppressing the RAS/ERK pathway
Source: Commun Biol. 2022 Sep 15;5:974. doi: 10.1038/s42003-022-03941-5 (PMC9478096; doi:10.1038/s42003-022-03941-5)
Supplement: Supplementary file 3 — Description of Additional Supplementary Data [file 42003_2022_3941_MOESM3_ESM.pdf]

Description of Additional Supplementary Files

File name: Supplementary Data 1

Description: The source data behind the graphs in Figure 2, Figure 3, Figure 4, Figure 5, and Figure 6 of the paper
