# Supplementary material for: Tmsb10 triggers fetal Leydig differentiation by suppressing the RAS/ERK pathway
Source: Commun Biol. 2022 Sep 15;5:974. doi: 10.1038/s42003-022-03941-5 (PMC9478096; doi:10.1038/s42003-022-03941-5)
Supplement: Supplementary file 5 — Reporting Summary [file 42003_2022_3941_MOESM5_ESM.pdf]

nature portfolio

Corresponding author(s): Ken-ichirou Morohashi

Last updated by author(s): Aug 30, 2022

Reporting Summary

Nature Portfolio wishes to improve the reproducibility of the work that we publish. This form provides structure for consistency and transparency in reporting. For further information on Nature Portfolio policies, see our Editorial Policies and the Editorial Policy Checklist.

Statistics

For all statistical analyses, confirm that the following items are present in the figure legend, table legend, main text, or Methods section.

n/a	Confirmed	
☐	☒	The exact sample size (*n*) for each experimental group/condition, given as a discrete number and unit of measurement
☐	☒	A statement on whether measurements were taken from distinct samples or whether the same sample was measured repeatedly
☐	☒	The statistical test(s) used AND whether they are one- or two-sided<br>*Only common tests should be described solely by name; describe more complex techniques in the Methods section.*
☒	☐	A description of all covariates tested
☐	☒	A description of any assumptions or corrections, such as tests of normality and adjustment for multiple comparisons
☐	☒	A full description of the statistical parameters including central tendency (e.g. means) or other basic estimates (e.g. regression coefficient) AND variation (e.g. standard deviation) or associated estimates of uncertainty (e.g. confidence intervals)
☐	☒	For null hypothesis testing, the test statistic (e.g. *F*, *t*, *r*) with confidence intervals, effect sizes, degrees of freedom and *P* value noted<br>*Give P values as exact values whenever suitable.*
☒	☐	For Bayesian analysis, information on the choice of priors and Markov chain Monte Carlo settings
☒	☐	For hierarchical and complex designs, identification of the appropriate level for tests and full reporting of outcomes
☒	☐	Estimates of effect sizes (e.g. Cohen's *d*, Pearson's *r*), indicating how they were calculated

Our web collection on statistics for biologists contains articles on many of the points above.

Software and code

Policy information about availability of computer code

Data collection	Bio-Rad Image Lab software (version 6.0.0) was used to collect data for the protein quantification from western blotting results. Bowtie 2 (version 2.3.4.1) and HTSeq (version 0.9.1) were used for alignment and assembly of the Single cell RNA-Seq read, respectively.
Data analysis	RStudio (Version 1.1.453 and 1.2.5033) with R (Version 3.4.3 and 3.6.3) was used for analyzing Single cell RNA-Seq data, generating bar graphs, and analyzing data statistically. Microsoft Excel (Version 16.59) was used to generate dot graphs and analyze data statistically.

For manuscripts utilizing custom algorithms or software that are central to the research but not yet described in published literature, software must be made available to editors and reviewers. We strongly encourage code deposition in a community repository (e.g. GitHub). See the Nature Portfolio guidelines for submitting code & software for further information.

Data

Policy information about availability of data

All manuscripts must include a data availability statement. This statement should provide the following information, where applicable:

- Accession codes, unique identifiers, or web links for publicly available datasets
- A description of any restrictions on data availability
- For clinical datasets or third party data, please ensure that the statement adheres to our policy

Single cell RNA-Seq data generated in this project are available from the DNA Data Bank of Japan with accession number DRA013467. Figure 1, Supplementary

Figure 1 and Supplementary Table 1 have associated source data included. Raw data in the figures or supplementary figures are available upon reasonable request to the corresponding author. Correspondence and requests for those materials should be addressed to K-I.M.

Human research participants

Policy information about studies involving human research participants and Sex and Gender in Research.

Reporting on sex and gender	*Use the terms sex (biological attribute) and gender (shaped by social and cultural circumstances) carefully in order to avoid confusing both terms. Indicate if findings apply to only one sex or gender; describe whether sex and gender were considered in study design whether sex and/or gender was determined based on self-reporting or assigned and methods used. Provide in the source data disaggregated sex and gender data where this information has been collected, and consent has been obtained for sharing of individual-level data; provide overall numbers in this Reporting Summary. Please state if this information has not been collected. Report sex- and gender-based analyses where performed, justify reasons for lack of sex- and gender-based analysis.*
Population characteristics	*Describe the covariate-relevant population characteristics of the human research participants (e.g. age, genotypic information, past and current diagnosis and treatment categories). If you filled out the behavioural & social sciences study design questions and have nothing to add here, write "See above."*
Recruitment	*Describe how participants were recruited. Outline any potential self-selection bias or other biases that may be present and how these are likely to impact results.*
Ethics oversight	*Identify the organization(s) that approved the study protocol.*

Note that full information on the approval of the study protocol must also be provided in the manuscript.

Field-specific reporting

Please select the one below that is the best fit for your research. If you are not sure, read the appropriate sections before making your selection.

☒ Life sciences ☐ Behavioural & social sciences ☐ Ecological, evolutionary & environmental sciences

For a reference copy of the document with all sections, see nature.com/documents/nr-reporting-summary-flat.pdf

Life sciences study design

All studies must disclose on these points even when the disclosure is negative.

Sample size	Sample sizes for each experiment are included in the legend or explained in the material and methods section.
Data exclusions	Cells in the G1 cell cycle phase were selected to avoid potential confounding effects from cell cycle-induced differences, and filtering was performed as described in the Methods section to exclude cells of low quality.
Replication	All data shown were replicated independently with the representative of n>=3 replicates. Details are explained in the legend and the methods section.
Randomization	The cells for each replicate were prepared on different days or from different sources. In our comparisons of single cell RNA-seq methods, we used essentially identical aliquots from the same source of cells.
Blinding	In all experiments, the investigator was not blinded to the treatment. Furthermore, all the analyses of this experiment are done by software or computer algorithms and should not be subjected to investigator bias.

Reporting for specific materials, systems and methods

We require information from authors about some types of materials, experimental systems and methods used in many studies. Here, indicate whether each material, system or method listed is relevant to your study. If you are not sure if a list item applies to your research, read the appropriate section before selecting a response.

Materials & experimental systems

n/a	Involved in the study
☐	☒ Antibodies
☐	☒ Eukaryotic cell lines
☒	☐ Palaeontology and archaeology
☐	☒ Animals and other organisms
☒	☐ Clinical data
☒	☐ Dual use research of concern

Methods

n/a	Involved in the study
☒	☐ ChIP-seq
☒	☐ Flow cytometry
☒	☐ MRI-based neuroimaging

Antibodies

Antibodies used	Please consult Supplementary Tables 4 and 6 for lists of all primary and secondary antibodies used.
Validation	The vendor validated all antibodies for immunostaining and western blotting analysis. Furthermore, the antibodies used for immunofluorescence were validated extensively in the publication cited in the Methods section (Sawano et al., 2019).

Eukaryotic cell lines

Policy information about cell lines and Sex and Gender in Research

Cell line source(s)	HEK293 cells (ATCC) were cultured in DMEM (Nacalai Tesque) supplemented with 10% fetal bovine serum and penicillin and streptomycin at 37 °C under 5% CO2. Cells were passed for at least two generations after cryorecovery before they were used for the assay.
Authentication	We did not perform cell line authentication.
Mycoplasma contamination	The cell line was mycoplasma negative.
Commonly misidentified lines (See ICLAC register)	None of these misidentified lines were used.

Animals and other research organisms

Policy information about studies involving animals; ARRIVE guidelines recommended for reporting animal research, and Sex and Gender in Research

Laboratory animals	Female ICR mice were bought from Japan SLC Inc. at six weeks old. We generated FLE-EGFP mice (Shima et al., 2012). In this study, FLE-EGFP male mice were crossed with ICR females, and we used the FLE-EGFP mice at E16.5.<br>In addition, we used ICR mice at E16.5 bought from Japan SLC Inc for in vitro testis reconstruction.
Wild animals	This study did not involve wild animals.
Reporting on sex	We used testicular cells from male mice at E16.5.
Field-collected samples	This study did not involve samples collected from the field.
Ethics oversight	All mouse experiment protocols were approved by the Animal Care and Use Committee of Kyushu University (Fukuoka, Japan).

Note that full information on the approval of the study protocol must also be provided in the manuscript.
